# The Differential Diagnosis of Gastric Ulcer, Chronic Gastritis and Gastric Cancer

**Published:** 1886-06

**Authors:** A. R. Davidson

**Affiliations:** Professor of Medical Chemistry and Toxicology, Medical Department, Niagara University; one of the Physicians to the Buffalo Hospital, Sisters of Charity


THE BUFFALO MEDICAL AND SURGICAL JOURNAL

VOL. XXVI. JUNE, 1886. NO. 11.

### Original Communications.

### *THE DIFFERENTIAL DIAGNOSIS OF GASTRIC ULCER, CHRONIC GASTRITIS AND GASTRIC CANCER.

BY A. R. DAVIDSON, M. D.,

Professor of Medical Chemistry and Toxicology, Medical Department, Niagara University; one of the Physicians to the Buffalo Hospital, Sisters of Charity.

There is no organ of the body whose well-being and efficacy is more important to the general economy than the stomach. I have hoped, therefore, that a rapid review of the etiology, symptoms and progress of the diseases named in the title of my paper (and to which I would add, as having symptoms in common, duodenal ulcer), and a discussion of those features which aid us in forming a differential diagnosis, might not be unprofitable. While all of them have features in common, yet, if we consult the carefully-compiled category of symptoms which most of the authors give us, it would appear that we ought easily to distinguish between them. Clinically, the very opposite is true, and, as an illustration, I venture to report a case, which, though it is not of the kind that we

* Read before the Buffalo Medical Club.

commonly hear from members of the club, may, perhaps, be not the less instructive.

A. B., aged 40, male, presented himself with the following history: Had led a roving life, with his full share of accompanying hardships and discomforts; not intemperate, but could enjoy an occasional glass. Had for the last six years suffered from occasional attacks of indigestion, and the symptoms during the last three years had been greatly aggravated, and steadily increased in severity. He complained of almost constant pains in the abdomen, locating it chiefly at the umbilicus, though, when severe, it radiated downward to the hypogastric and right inguinal region. He had a good appetite; but commonly, after eating (not immediately, but from one to two hours afterward), he had an attack of pain and burning in the epigastrium, with the shooting pain before noted, which often became intolerable. He then relieved himself by tickling his throat with his finger, thereby causing himself to vomit; emesis never occurred spontaneously. In the morning he frequently had acid eructations; but as he said, "I never vomit unless I put my finger down my throat, and I can always make myself vomit by doing that."

The ejections were always strongly acid. He had never vomited blood. From his frequent changes of residence he had consulted many physicians, and he said they all told him he had chronic dyspepsia. Before calling upon me, he had been treated by an excellent physician, who had put him on a diet limited to peptonized milk. He left him because, as he expressed it, "I would rather die than be starved to death. I am growing weaker every day, and suffer just as much pain as when I eat something nourishing." To my expression of opinion that it was good treatment, and that he had probably had no occasion to produce emesis since following it, he asserted that the milk was not only exceedingly distasteful to him, but that if he drank any quantity of it he was sure to have pain, and he had to relieve himself quite as frequently as before commencing the use of the peptonized milk. The milk, when rejected, was curdled and stringy, but not colored. He was positive he had had no black vomit, nor had he ever vomited blood. In answer to the question whether his stools were black, he replied in the negative, but complained of persistent constipation, and to this he attributed all his troubles. As to family history: One sister had been troubled as he was, and had died. The doctors had said she had a tumor. There was no *post-mortem*.

A physical examination showed abdomen tympanitic and stomach distended; no pain could be produced, even by deep pressure. A greater resistance to the finger than is usual was thought to be detected over the pylorus, but no definite signs of a tumor could be discovered. Liver and heart normal; dullness at apex of right lung; skin very pale, with a yellowish cast; great emaciation and marked feebleness; indeed, the walking of a few blocks was all that he could do.

I have given you all the diagnostic points I was able to elicit, except that an examination of the urine showed a deficiency of urea, otherwise the fluid was normal. Perhaps there is enough here for some of you to make a diagnosis, but there was not for me. It might be chronic gastritis, gastric cancer or ulcer, or duodenal ulcer. Sufficient for the present to say that I leaned to the probability of the case being one of chronic gastritis with some pyloric stenosis, or duodenal ulcer. The results from the treatment adopted seemed to justify that opinion.

The man made several calls at my office during the succeeding three weeks, and his confidence in his daily improvement seemed to be justified by his appearance. He had had but few attacks of pain, and those of a milder form. He had gained strength, he no longer suffered from hunger, constipation was greatly relieved, and his despondency was exchanged for hope.

One night, at the end of the third week, I was called to his house. I found him in an agony of pain. I gave him a half grain of morphia hypodermically, and in a half an hour, as this was insufficient, a quarter more; this gave him some relief. When I left him I gave him a few quarter-grain granules, to be taken if the pain again became unendurable. In the morning I found him free from pain. He would flinch upon placing the hand lightly upon his abdomen, but did not complain upon pressure. He was perfectly conscious; skin cool; extremities cold; face pale and pinched; pulse 120, and weak; and— I advised the family to lose no time in summoning his spiritual adviser. In the evening he was dead.

*Post-mortem* revealed a perforating ulcer on the minor curvatures near the pyloric end of the stomach. There was some thickening of the sub-mucous and muscular coats, but not enough to cause obstruction of the pyloric orifice; liver healthy; no obstruction nor lesion in the intestines. Tubercular deposits in the apices of both lungs, more extensive in the right.

As another illustrative case, let me quote one reported by Dr. Flint, Sr.:

Miss P., aged about 30, was seen by him in consultation. She was greatly emaciated; had suffered much from abdominal pain, and had been led to the use of morphia, taking about a grain daily. She had dyspeptic ailments, but the usual diagnostic symptoms of gastric ulcer were wanting.

A diagnosis was not made. She was advised to discontinue the use of morphia, and take nutritious food, with tonic remedies. She improved in a marked degree in the following few weeks, when she was suddenly seized with intense pain in the abdomen, tympanites, and prostration.

She died the following day. The autopsy revealed the existence of an ulcer, with perforation just below the pylorus.

Let us now consider the etiology, symptoms and progress of these affections separately, and then attempt a comparison of their prominent characteristics:

### GASTRIC CANCER.

Cancer is found more frequently in the stomach than in any other organ except the uterus. Loomis gives the varieties in the order of their frequency as follows: (1) Scirrhus, (2) Medullary, (3) Colloid, (4) Villous, (5) Melanotic, (6) Epithelial. The last three varieties are exceedingly rare. In fact, clinically, we can make no such fine distinction. What is called cancer of the stomach means, in the large majority of cases, scirrhus.

In three-fifths of the cases it has its seat at the pyloric extremity of the stomach. It occurs most frequently between the ages of 40 and 60. Welch's table of 2,038 cases shows 2 under 20 years of age, 55 under 30, 271 between 30 and 40, 499 between 40 and 50, 620 between 50 and 60, 428 between 60 and 70, 140 between 70 and 80, 20 between 80 and 90, 3 over 90.

Hereditary predisposition is asserted by nearly all the authors as its most important etiological factor. Lebert denies this. In 52 cases only 5 had such history, and considering the frequency of the disease, it is not to be wondered at if it should appear in two or more members of the same family.

*Symptoms and Progress.*—Cancer of the stomach may exist in a latent state; that is, an advanced cancerous deposit may be seated in the gastric organ without exhibiting any of its peculiar symptoms, but, in the majority of cases, before the characteristic tumor is felt in the epigastrium, other phenomena, more or less disturbing in character, attract attention.

Most prominent among the early symptoms is anorexia, accompanied by a sense of uneasiness or distention in the epigastrium, with nausea and vomiting; intense acidity and sour eructations are very common. Later the more characteristic symptoms appear, as the shooting pain, often referred to the right or left hypochondrium, the cancerous cachexia, and the melanic vomit. Let us consider these symptoms separately.

*Appetite.*—Most of the authors agree in stating that the appetite is impaired; others, while admitting that this is generally true, assert that it is occasionally normal or exaggerated.

*Vomiting.*—This symptom is seldom wanting. Lebert observed it in 24 cases out of 26, while Brinton's tables show this symptom present in 87 per cent. of the cases. The matter vomited usually contains blood. Profuse hæmatemesis is rare.

The blood vomited is usually changed by the gastric fluid, presenting the well-known "coffee-ground" appearance. Of this symptom, Bristow says: "It is mostly caused by the taking of food, and comes on at different periods after it. If the cardiac orifice be contracted, the food is usually returned at once by regurgitation; if the pylorus be affected, the vomiting is often delayed for an hour or two, or more. When the stomach is irritable, vomiting may take place almost immediately after food has entered the stomach."

*Tumor.*—In the epigastrium may or may not be discovered. If the cardiac orifice or cardiac extremity of the stomach is the portion affected, it is altogether unlikely that a tumor can be detected, however large or extensive. One situated at the pyloric extremity will be probably recognized; though if small, deep-seated and elastic, it may readily escape detection.

*Cachexia.*—The supposed characteristic straw-yellow color of cancer is not often met with, says Da Costa. Loomis, Flint and others speak of it as usually present.

*Hiccough, flatulence and constipation* are commonly very annoying. *Diarrhœa* may appear in the later stages; that is, when ulceration has occurred, the cancerous detriti, pus, etc., acting as the exciting cause.

As to the *pyretic symptoms* the authors differ. In exceptional cases doubtless a moderate amount of irritative fever accompanies the general wasting. According to Brinton, fever appears more frequently than is commonly supposed, the tongue coated with a whitish, thick fur, and the eyes red, the face flushed, the urine scanty and overloaded with urates and urea. As to the condition of the urine, he is directly opposed by later observers.

CHRONIC GASTRITIS, known also under the names of simple gastritis, chronic catarrh of the stomach and chronic inflammatory dyspepsia.

As to the etiology of the disease, I cannot give you a better summary than that of Loomis: "It is essentially a secondary affection; it is rarely the sequela of sub-acute, much less of acute gastritis, unless the former has been caused by an abuse of alcoholic stimulants. In many persons there is a hereditary tendency after middle life to chronic gastric catarrh. The principal, general cause is anæmia. The most common local cause is the daily use of alcoholic stimulants. Mechanical obstruction to the capillary circulation of the stomach, inducing continued passive hyperæmia, will cause it, and hence we find it associated with cirrhosis of the liver, and other chronic hepatic affections, where the blood is dammed back in the formative branches of the vena portæ. In the same way cardiac and pulmonary diseases, which offer an obstacle to the venous return, will induce chronic gastritis. Pressure on the walls of the stomach by tumors produces first congestion, and then chronic catarrh. Degeneration of the capillaries, occurring in the cirrhotic form of Bright's disease, causes it, and it often also accompanies ulcer and cancer of the stomach."

*Symptoms and Progress.*—The early symptoms are chiefly those of indigestion. The patient complains of a sense of weight, constriction or heat. Later there is frequently severe pain and burning in the epigastrium. This condition may remain stationary for some time, may terminate in recovery, or become aggravated. In the latter case the pain becomes acute and often continuous, and often described as lancinating. It is usually increased after eating, or by pressure.

*Appetite.*—May be impaired or exaggerated, and again there may be complete anorexia.

*Nausea.*—Is one of the most frequent symptoms.

*Vomiting.*—Is not usually present. Loomis says that the most important of the dyspeptic symptoms are the acid risings after meals, and the vomiting or regurgitation of acid mucus in the morning, without which he considers the diagnosis uncertain. If there is actual vomiting of food, traces of butyric acid are present with the sarcinæ ventriculi. Black vomit has been observed in some cases in the last stages of the disease. Hæmatemesis often occurs in that form of gastritis that accompanies cirrhosis of the liver. If hæmorrhagic erosion exists in a stomach, which is the seat of gastric catarrh, all of the symptoms of gastric ulcer may be present.

*Constipation.*—Is very common, but it may alternate with diarrhœa from extension of the inflammation to the intestinal tract. Gastritis is a disease of lingering and irregular course, and may terminate in ulcer or stenosis of the pyloric orifice. Death may result from hæmatemesis or from inanition, but the general feebleness which results from long standing gastritis, predisposes to acute diseases.

### GASTRIC ULCER.

All the determining causes of chronic inflammation of the stomach, says Cruvelhier, can provoke the development of the simple ulcer. Leudet and Niemeyer assert that the excessive use of alcoholic liquors is one of the most frequent causes. This is denied or not referred to as an etiological factor by many authors. It is generally associated with anæmia, or follows chronic gastric catarrh, or embolic plugging of small arterial twigs, or other disturbances of the circulation in the gastric mucous membrane. The habitual stooping position, as in various trades, has been considered as causative. It is said to be frequently met with in connection with a cirrhotic kidney. It has been supposed to have some connection with the puerperal state, lactation, hæmorrhoidal affection, or menstrual disturbances. Cartheurt-Sees named it menstrual ulcer. It is certainly more common in females than in males. Brinton, in 650 cases, observed 440 in women; Jaksch, in 340 cases, reports 232 in women. All ages are liable to it, but the following table gives the age in 607 cases of open ulcer, and proves the greater frequency of the disease after adult age:

| Ages, . . . . . . . . . . | 1–10 | 10–20 | 20–30 | 30–40 | 50–60 | 60–70 | 70–80 |
|---|---|---|---|---|---|---|---|
| Number of cases, . . . . . | 1 | 32 | 119 | 107 | 108 | 84 | 35 |

Over 80 years of age several cases are reported.

As regards its nature, there are the usual divergent views. Grisolle, Hardy and Behier consider it an ulcerous chronic gastritis; Rokitanski, as a circumscribed gangrene from unknown causes; and Cruvelhier believes it to be due to an inflammation of the mucous follicles. Virchow explains the formation of the ulcer by an arterial embolism resulting in an eschar within the limits of the affected vessels, which, acted upon by the gastric juice, disappear, leaving an ulcer in its place.

*Symptoms and Progress.*—The diagnosis of the disease must be made upon symptoms directly referable to the stomach; pain, tenderness upon pressure, vomiting and hemorrhage are the important symptoms, but they vary greatly in their maniestations. Da Costa says that the peculiarities the pain exhibits, forms, upon the whole, the most distinctive symptoms of gastric ulceration, and he gives them as follows: "It is generally a continuous dull feeling, sometimes a burning, sometimes a gnawing sensation. As a rule, it is rendered more acute within a quarter of an hour after eating, and remains as long as food occupies the stomach. Its situation is commonly in the middle of the epigastric region, and is there strictly limited. At that point, too, there is localized soreness, or even great tenderness to the touch. Sometimes the pain is seated behind the ensiform cartilage, or is referred to the right or left hypochondrium. It is often associated with a gnawing pain in the lower dorsal vertebra, which may shoot between the scapulæ or down the spine; but the dorsal pain, like the epigastric, is, on the whole, very fixed, radiates but little, and is most severe when the ulcer is on the posterior surface. Besides this continued feeling of distress, there occur violent paroxysms of pain, which may last for several hours, or, with trifling intermission, for days. They are aggravated by pressure, or by food; in fact, they are often thus induced, but not always, for they sometimes come on suddenly when the viscus is empty. The patient refers the suffering chiefly to the pit of the stomach, or to the dorsal vertebræ. He is apt to seek the recumbent position for relief. Yet it is not a little remarkable that there are sometimes long intervals during which all pain, whether paroxysmal or not, ceases, and during which food can be taken without inconvenience."

In every author's description of this symptom, there is more or less divulgence. The character of the pain seems to be commonly influenced by the location of the ulcer. In the majority of cases this is on the posterior wall of the stomach, in or near the lesser curvature, and towards the pyloric extremity. Osborne and Brinton hold that the influence of certain positions of the body, to exacerbate or calm the pain, throws light upon the location of the ulcer. "If the decubitus ventris is borne with comfort, the site of the ulcer is on the posterior wall; the right lateral position, without aggravation of pain, points to the ulcer in the opposite direction, and the same holds good as to the decubitus dorsalis." The tenderness upon pressure is by no means a constant sign, and is no doubt influenced by the location of the ulcer.

*Vomiting* may be absent from first to last. It usually comes on at some stage, is, and by most of the authors is said to be, very persistent. In its association with hemorrhage, it is generally considered as a symptom of high value in the differential diagnosis. It is the only kind of vomit at all distinctive of gastric ulcer, and if, with other symptoms, we have the occurrence of profuse hæmatemesis, there is little room for doubt if the patient be a male; but, as Da Costa points, vicarious hemorrhages are not at all unlikely to occur in young women who are troubled with amenorrhœa, and one of the marked traits of hysterical disorder is that it manifests itself by tenderness in the epigastric region, and by pain in the stomach. We thus may have the most significant sign of gastric ulcer occurring—as so many cases of amenorrhœa do—in chlorotic young females, the class among which ulceration of the stomach is most frequent.

As I have quoted from Da Costa's description of the symptoms, it is but fair to note that he also says that all of the phenomena may be met with in other disorders, and that many a case of ulceration of the stomach occasions nothing but the symptoms of chronic gastritis; and, again, that symptoms almost identical with gastric ulcer may be produced by *duodenal ulcer.* This affection, he says, if more frequent, would be a constant source of error. Recent investigations would seem to show that ulcers of the duodenum are of much more frequent occurrence than has been supposed. Thus, according to Niemeyer, duodenal ulcers or their cicatrices were found by Willigk seventy-six times in one thousand *post-mortems*, in two cases the ulcers being perforating. They are usually situated in the upper horizontal portions, and are more common in men than in women.

Let us now proceed to compare the symptoms common to the four affections. Some of the authors do this for us, and by grouping the prominent characteristics of each together, they bring out differences which apparently make their differential diagnosis easy.

Take, for instance, the table given by Da Costa in his work on Medical Diagnosis:

| CHRONIC GASTRITIS. | GASTRIC ULCER. | GASTRIC CANCER. |
|---|---|---|
| 1. Pain at the epigastrium somewhat augmented by food; also soreness. Both constant, although comparatively slight. | Pain at the epigastrium much augmented by food; subsides when this is digested; paroxysms of pain, but not lancinating; a strictly-localized soreness to the touch in the epigastric region, sometimes a painful spot over the lower dorsal vertebræ. Intermissions in the pain of considerable length are frequent. | Pain frequently of a radiating kind, often paroxysmal, not unusually severe and lancinating, but not of necessity associated with soreness. Little or not at all affected by food. Pain rarely remits; never intermits for any considerable time. |
| 2. Symptoms of indigestion. | Symptoms of indigestion sometimes very slight. | Symptoms of indigestion. Anorexia; extreme acidity of stomach. |
| 3. Sometimes vomiting. | Vomiting may be present or absent. | Vomiting a very frequent symptom. |
| 4. No hemorrhage, or but trifling hemorrhage; and even a trifling hemorrhage is rare. | Abundant hemorrhage from the stomach common. | Hemorrhage not very abundant, but occasioning frequently coffee-ground-looking vomit. |
| 5. Bowels constipated | Bowels may or may not be constipated; usually are. | Bowels obstinately constipated. |
| 6. No fever. | No fever. | Attacks of moderate fever may occur. |
| 7. Not much emaciation; no cachectic appearance. | Frequently extreme pallor and debility. | Gradual and progressive loss of flesh, and debility; and at times with the cachexia hypertrophy of the peripheral lymphatic glands, especially above the clavicles. |
| 8. Not confined to any age. More common in middle-aged or elderly people. | May occur in middle-aged persons; but is also frequently seen in young adults, especially in young women. | Most common in elderly people; rarely occurs in persons under 40 years of age. |
| 9. Disease may be relieved or cured, or is of very long duration. | Duration uncertain; may get well, may run on rapidly to perforation, or may last for years. | Average duration 1 year; may be shorter, but seldom longer; very rarely reaches two years. |
| 10. No tumor. | No tumor. | Generally a tumor. |

To these I would add the following:

| | | |
|---|---|---|
| 11. Free hydrochloric acid may be present in the gastric contents. | Free hydrochloric acid usually present. | Free hydrochloric acid usually absent. |
| 12. Urinary examination; abundant deposit of urates common. | Urinary examination normal. | Urinary examination frequently shows marked diminution of urea, occasionally an excess of acetone and indican. |
| 13. Microscopic examination. Sarcinæ ventriculi sometimes present in cases of long standing. | Microscopic examination. Sarcinæ ventriculi (very rare). | Microscopic examination. Sarcinæ ventriculi not infrequent; fragments of cancerous tissue may be found in the ejecta from the stomach. (Rare.) |
| 14. Eructations from stomach; odor not abnormal | Eructations from stomach; odor not abnormal. | Eructation from stomach; a peculiar fetor. (Rare.) |
| 15. Causation often referable to abuse of alcohol, gormandizing, and certain diseases, as nephritis, phthisis, cirrhosis of | Causation unknown. | Causation unknown. |

Such a table is of value simply as showing the differences derived from an analysis of well-marked cases, but they do not hold good in the majority of them. It is the whole complex of symptoms, the order of their occurrence and the general aspect of the case, which must be carefully considered in forming a diagnosis.

The differential points mentioned are of very unequal value. Let us consider the symptoms in the order given in the table.

1st, *Pain.*—This, in all the comparative tables, is made a symptom of diagnostic value. That it is more apt to be augmented by pressure or food in gastritis and ulcer than in cancer is undoubtedly true, but it may not be prominent in ulcer, and may be marked in cancer, and may be absent in both. As to the character of the pain, whether acute or dull, burning or lancinating, very little dependence can be placed on the statements of patients. Lancinating pain is not distinctive of cancer; may be present in all. Strictly localized pain, xiphoidian and rachidian, of Cruvelhier, is not present in the majority of cases of ulcer.

Pain on pressure may be present in all; intermissions in the pain are common to all.

*Symptoms of Indigestion* are common to all, as also acidity and changes in appetite, and vomiting.

*Hemorrhage.*—When abundant, a sign of great importance in ulcer, but is not pathognomonic; may be present to some degree in all; coffee-ground vomit not distinctive of cancer.

*Constipation.*—A symptom of no value differentially.

*Fever.*—Pyretic symptoms are given by different authors as present in all forms of gastric disturbance.

*Cachexia.*—Quesnels asserts that every cachectic person suffering from a chronic affection of the stomach has carcinoma. Such a proposition cannot be maintained. The so-called cancer cachexia is not often met with. We may have a cachectic appearance with gastritis and ulcer.

*Age.*—Is of value only in probably excluding cancer in young persons. Mathieu draws attention to the fact that cancer of the stomach may occur in early life, and reports 27 cases occurring before the age of 30, and in every instance the *post-mortem* proved a wrong diagnosis had been made.

*Duration.*—All of the authors agree in stating that the duration of carcinoma is shorter than that of other chronic affections of the stomach, but there are exceptions to this. Dujardin-Beaumetz cites one case of his own, in which the symptoms preceded death by twelve years.

*Tumor.*—An epigastic tumor is generally accepted as establishing the diagnosis of a cancer, but this, if present, is often extremely difficult to find, chiefly because of its mobility in relation to the condition of the stomach and of its adhesions. The German authorities advise, as an aid to diagnosis, the distension of the stomach by liquids or carbonic acid gas. Beaumetz asserts that this procedure is of no value; but a tumor, if found (unless, indeed, it be well defined and lobulated), does not certainly establish the diagnosis. A marked thickening of the pyloric extremity may occur in gastritis. Trier speaks of a tumor in the epigastrium as common in duodenal ulcer resulting from adhesion with the pancreas. Again, a cancerous enlargement of the pancreas may present all the physical characteristics of cancer of the stomach, or a cancer of the left lobe of the liver. In deciding that a tumor in the epigastrium is referable to the stomach, its association with gastric symptoms is, of course, important; but it is to be remembered that in cancer of the stomach gastric symptoms are sometimes wanting, and tumors of neighboring organs are frequently accompanied with derangements of the stomach. The discovery of secondary cancers in the liver, in the peritoneum, or in the lymphatic glands, may render valuable aid in diagnosis.

As to the other differential points which I have added, Vandervelder asserts that in the case of cancer hydrochloric acid is absent in the gastric juice, and always present in gastritis or ulcer. The test is applied by causing gelatine capsules, containing sponge attached to a thread, to be swallowed. In about fifteen minutes they are withdrawn, and the fluid obtained subjected to examination.

Kredel, with an experience of nineteen cases, considers this test of great value. Ewald, Beaumetz and others report cases in which free hydrochloric acid was present in stomachs dilated from gastric cancer. While this symptom cannot be considered as pathognomonic, the presumption would be against gastric cancer if hydrochloric acid be found continuously in a dilated stomach. Trousseau considers painful œdema of the legs as characteristic of cancer. This is emphatically denied by many. Romelaere, of Brussels, declares that in gastric cancer the amount of urea excreted is greatly diminished, falling as low as 120 to 150 grains in twenty-four hours, while in gastric ulcer it never falls below 225, but varies from that up to 500. Dujardin-Beaumetz found Romelaere's statement to be correct in a number of instances, and, influenced by this, finding forty-five grains of urea to be daily voided by a patient with gastric symptoms, made a diagnosis of cancer of the stomach. The necropsy showed it to have been a hydatid cyst of the liver.

Nevertheless, I believe that Romelaere's method has value. There is, without doubt, an impoverishment of nutrition, and, consequently, lessened excretion of urea, associated with malignant tumors, that is not observed in connection with the benign. But it must not be forgotten that gastric ulcer and gastric catarrh are frequently associated with cirrhotic kidney. The urinary examination will also frequently show an excess of indican and of acetone. Acetonuria has been observed especially in diabetes mellitus, but it occurs also in cases of gastric cancer, and may or may not be associated with the peculiar form of coma which has been described by Kussmaul as distinguishing diabetic coma.

Another symptom of gastric cancer, which is not mentioned in the books, may be considered as pathognomonic if present, viz., *a peculiar fetor to the eructation.* Fetor of the breath is spoken of in some of the text-books, but this may arise from so many causes that no reliance can be placed upon it as a means of diagnosis. But fetor of the eructation is from the contents of the stomach. If it has not simply a putrefactive, but a cancerous odor,—a smell which cannot be described, but is familiar enough to those who have treated cancer of the uterus,—then we have a pathognomonic symptom upon which you can make a diagnosis. Still it is true that the majority of cancerous stomachs opened after death have no cancerous odor, and during life have no fetid eructations.

Another pathognomonic symptom is sometimes to be obtained by the microscope. Fragments of cancerous tissue may occasionally be found in the ejecta. Their absence would not disprove the existence of cancer; their presence would settle the question without a doubt.

The presence of sarcinæ indicates that there is some condition which prevents the stomach from completely emptying itself. They are always indicative of structural change, therefore we are most apt to find them in cases of gastric cancer. They are not found in cases of gastritis or ulcer, unless a narrowing of the pyloric extremity has led to distention of the organ.

It has been hoped that with the advance now being made in electrical illumination we might receive valuable assistance in the diagnosis of obscure cases by the employment of the gastroscope. So far, however, it must be admitted that it has proved of little value, and its employment not without danger to the patient.

